# Low Level of Dietary Organic Trace Elements Improve the Eggshell Strength, Trace Element Utilization, and Intestinal Function in Late-Phase Laying Hens

**DOI:** 10.3389/fvets.2022.903615

**Published:** 2022-05-27

**Authors:** Xing Chen, Xiang-ming Ma, Chong-Wu Yang, Shu-zhen Jiang, Li-bo Huang, Yang Li, Fan Zhang, Ning Jiao, Wei-ren Yang

**Affiliations:** ^1^Department of Animal Sciences and Technology, Shandong Agricultural University, Tai'an, China; ^2^Dongying Science and Technology Innovation Service Center, Dongying, China; ^3^Guelph Research and Development Center, Agriculture and Agri-Food Canada (AAFC), Guelph, ON, Canada

**Keywords:** organic trace element, egg quality, utilization, intestinal function, laying hen

## Abstract

This study was conducted to evaluate the effects of organic trace elements (Cu, Fe, Zn, and Mn) on performance, egg quality, trace elements utilization, and intestinal function in late-phase laying hens. A total of 1,080 laying hens (Hy-line brown, 65 weeks old) were randomly assigned to four treatments with six replications of 45 layers each. The basal diet was prepared without adding exogenous trace elements. The control group was fed with a basal diet supplemented with 600 mg/kg of inorganic trace elements. The three treatment groups were fed basal diets supplemented with 300, 450, and 600 mg/kg organic trace elements (OTE300, 450, and 600), respectively. The results showed that there was no significant difference in growth performance among all treatments. However, OTE450 significantly improved the eggshell strength of laying hens (*p* < 0.05), but had no significant effects on haugh unit, egg yolk weight, eggshell weight, and eggshell thickness, compared with other groups. Moreover, compared with the control group, OTE450 significantly increased the contents of copper, iron, and zinc in serum (*p* < 0.05). Meanwhile, all of the trace elements had a lower deposition in the feces in organic trace elements groups (*p* < 0.05). Histological analysis showed that the addition of organic trace elements could significantly improve the villus height and villus concealment ratio (*p* < 0.05). In addition, the messenger RNA (mRNA) and protein expressions of divalent metal transporter 1 (*DMT1*), zinc transporter 1 (*ZnT-1*), and ferroportin 1 (*FPN1*) were the highest in the OTE450 group. In conclusion, OTE450 could improve egg quality, intestinal function, and trace element utilization efficiency. Thus, this study provides a theoretical basis for the application of low levels of organic trace elements in laying hens.

## Introduction

Trace elements, as coenzyme factors of many metal enzymes, play important roles in digestion, absorption, metabolism, immune response, and biosynthesis (1, 2). To meet the requirement of animals, inorganic trace elements were commonly supplemented in diets. However, inorganic trace elements are easy to interact with other compounds to form insoluble substances, resulting in a high supplemental amount in feed following low absorption efficiency in the intestine and more excretions into the environment (3, 4). Organic trace elements are complexes or chelates formed by inorganic trace elements and organic complexes, such as proteins, amino acids, organic acids, small peptides, polysaccharides, and derivatives. Organic trace elements have higher bioavailability than inorganic trace elements because they can be absorbed through other transport routes, such as amino acid transport channels (5). The study reported that dietary supplementation of organic trace elements could improve egg quality and intestinal health and increase serum mineral element levels (6). In addition, compared with inorganic trace elements, a lower level of organic trace elements in feed can improve the eggshell quality, antioxidant capacity, and immune function and reduce the excretion of mineral elements in feces without impacting animal production performance (7–9). It has been reported that compared with traditional inorganic form, dietary supplementation of amino acid chelated trace elements, such as copper, iron, zinc, and manganese could significantly improve chicken growth performance, antioxidant capacity, and trace element absorption and decrease mineral excretion (9–13).

Low levels of organic compound trace elements improve the eggshell quality, antioxidant capacity, immune function, and mineral deposition of aged laying hens (14). In the late stage of laying, the strength and thickness of the eggshell and the ability of hens to transport and absorb trace elements are reduced (15). Most studies focus on the effects of single trace element supplementation on late-phase laying hens (16, 17).

However, few studies focus on the effects of organic trace elements on late-phase laying hens. Moreover, there are few reports on the study of duodenal trace element transport. The purpose of this study was to study the effects of organic trace elements on laying performance, egg quality, trace element deposition, intestinal morphology, and related trace element transporters, and to find the optimal amount of organic trace elements for late-laying hens' production performance.

## Materials and Methods

### Experimental Design and Layers Management

The layers used in this experiment were cared for in accordance with the guidelines for the care and use of laboratory animals described by the Guide for the Care and Use of Laboratory Animals and approved by the Committee on the Ethics of Shandong Agricultural University (ID: S20180058).

A total of 1,080 Hy-line brown laying hens with the same genetic background and comparable body weight (1.55 ± 0.02 kg) aged 65 weeks were arranged in metal cages (40 cm ×45 cm × 45 cm, Shandong Renhe mechanical equipment Co., Ltd, Taian, China). The temperature of the chicken house was controlled between 20 and 25°C, and the chicken house was exposed to light for 16 h every day. The layers were randomly divided into four treatments, each treatment with six replicates and 45 layers per replicate for a 10-week feeding trial following a 7-day adaptation. Layers were allowed *ad libitum* access to feed and water. The health status and mortality of laying hens were recorded in time. Throughout the trial period, egg number and egg weight were recorded every day. Egg-laying rate, average daily feed intake, and feed egg ratio were calculated every week in replicates.

The experimental diets were formulated according to NRC (18) except that the form of trace element was provided as an experimental design. The composition and calculated nutrient levels in the basal diet are shown in Table 1. The control group was fed with a basal diet supplemented with 600 mg/kg of inorganic trace elements, and all other groups were fed with a basal diet supplemented with 300, 450, and 600 mg/kg organic trace elements (OTE300, 450, and 600) (Fujian ShenNa Bioengineering Co., Ltd., Fujian, China). The organic trace elements used in this study were coated with hydroxymethyl cellulose and starch. The levels of inorganic trace elements and organic trace elements in diets are shown in Table 2. Before the 10-week trial period, a 1-week adaptation was used to change the commercial diet into an experimental diet.

**Table 1:** Dietary components and nutrient levels in the basal diet (as fed-basis).

**Ingredients**	**Content (%)**	**Nutrient levels**	**Contents**
Corn	61.00	Metabolisable energy (MJ/kg)	11.15
Soybean Meal	25.00	Crude Protein, %	15.53
Soybean Oil	1.00	Calcium, %	3.78
Limestone	8.00	Phosphorus, %	0.58
Premix^a^	5.00	Non-phytate Phosphorus, %	0.32
Total	100.00	Methionine, %	0.35

a *The premix provided the following per kilogram of diet: sodium chloride 3.2 g, vitamin A 7,000 IU, vitamin D_3_ 2,250 IU, vitamin E 20 IU, vitamin K_3_ 1.13 mg, vitamin B_1_ 1.68 mg, vitamin B_2_ 6 mg, vitamin B_3_ 45 mg, vitamin B_5_ 10 mg, vitamin B_6_ mg, vitamin B_9_ 0.75 mg, biotin 0.11 mg, and vitamin B_12_ 0.01 mg*.

**Table 2:** The forms and levels of trace elements in diets (mg/kg).

**Element**	**Control**	**OTE300**	**OTE450**	**OTE600**
Copper	7.80	3.90	5.85	7.80
Iron	84.00	42.00	63.00	84.00
Zinc	72.00	36.00	54.00	72.00
Manganese	72.00	36.00	54.00	72.00
Selenium	0.48	0.24	0.36	0.48
Iodine	0.48	0.24	0.36	0.48

### Sampling Collection

At week 10, two laying hens with similar body weight (BW) were selected in each repeated and fasted for 12 h. After blood collection from the brachial wing vein, the laying hens were humanly sacrificed. The serum was stored at −20°C until analysis.

At the end of the experiment, feces were collected for 3 consecutive days with a feces collection board. Feces collected every 24 h were stored in polyethylene bags. The collected feces were dried at 65°C for 48 h and then dried at 105°C. After crushing, it passes through a 1 mm screen, the feces were stored at −20°C.

The duodenum was isolated under sterile conditions after slaughter, and part of the duodenum was intercepted and stored at −80°C for gene and protein analysis. Moreover, approximately 3 cm of the middle duodenum was fixed in 4% (w/v) of paraformaldehyde for analyzing intestinal morphology and immunohistochemical analysis.

### Egg Quality Analysis

At the end of the experiment, six eggs in each replicate with similar size and weight were randomly selected to measure egg weight, albumen height, and yolk height with a full-automatic eggshell strength tester (EFG0503; Robot Machine Co., Ltd, Tokyo, Japan) and a multifunctional egg quality analyzer (EMT5200; Robot Machine Co., Ltd, Tokyo, Japan). In addition, the eggshell thickness was measured with an automatic eggshell thickness meter (Echometer, Model 1061; Robot Machine Co., Ltd, Tokyo, Japan). Moreover, eggshell and yolk weights were recorded.

### Serum and Feces Trace Elements Analysis

Copper, iron, zinc, and manganese in feces and serum were determined by inductively coupled plasma atomic emission spectrometry (ICP-AES, Optima 8300, Perkin Elmer, Waltham, Ma, USA). Briefly, the sample was ashed and carbonized, dissolved with a 6 mol/L hydrochloric acid solution, and fixed with water into a 50 ml volumetric flask to be measured.

### Duodenum Morphological Observation

The fixed duodenum was embedded in a fully automatic rotary paraffin slicer (HM355S, Burton International Trading Co., Ltd, China), sliced to a 5 μm thickness using a microtome device. After that, the samples were dyed by an automatic dyeing machine (A81500101, Thermo Scientific, UK) and sealed with neutral resin. The morphology of the duodenum was observed and photographed by microscope (Nikon elipse 80i, Japan). For each sample, 10 well-oriented villi and corresponding crypts were measured by an image analyzer (Lucia software, zadrahau), and the ratio of villi to crypts was calculated.

### Immunohistochemical Analysis

Duodenal sections were prepared according to the standard immunohistochemistry (IHC) protocols. After the sections were dewaxed, the antigen was repaired by microwave oven in sodium citrate buffer (0.01 mol/L, pH = 6) for 5 min each time. The sections were repeated three times, then treated with 10% hydrogen peroxide for 1.5 h, and then incubated in 10% goat serum (ZSGB-BIO, Beijing, China) for 1 h to block nonspecific binding. Immunohistochemical analysis was performed using a kit (Polink-2) plus^®^ Polymer HRP Detection system for rabbit primary antibody, PV-9001 (ZSGB-BIO, Beijing, China). After being washed with hydrochloric acid buffer, the above-prepared sections were incubated with antibodies Anti-*DMT1* (1:100, bs-3577R, BIOSS, Beijing, China), Anti-*ZnT-1* (1:100, bs-6440R, BIOSS, Beijing, China), and Anti-SLC40A1/*FPN1* (1:100, 26601-1-AP, Proteintech, Wuhan, China) at 4°C overnight. Then, the slices were washed with phosphate-buffered saline (PBS), incubated with primary antibodies at 37°C for 1 h, and then incubated with Polink-2 plus Polymer HRP anti-rabbit secondary antibodies at 37°C for 1 h. After incubation, the slices were washed with PBS, soaked in diaminobenzidine (DAB) with a kit (DAB kit, TIANGEN PA110, Beijing, China) for 2 min, tested for immunostaining, and finally sealed with neutral resin. The immunoreactive substances were located with a bright field of vision using a microscope. Three stained sections of each sample were randomly selected for observation and shooting, and the images were analyzed by image analysis software (imagepro Plus6.0, media control, Yinquan, MD, USA). The total cross-sectional integrated optic density (IOD) was obtained and the staining intensities of divalent metal transporter 1 (*DMT1*), zinc transporter 1 (*ZnT-1*), and ferroportin 1 (*FPN1*) between the control group and the coating treatment were compared. The IOD of each sample is the average of the three stained sections.

### Quantitative Real-Time PCR (qRT-PCR) Analysis

Total RNA was extracted from the duodenum of laying hens using Trizol total RNA extraction reagent according to the manufacturer's instructions. Total RNA was reverse transcribed into cDNA using a reverse transcription Kit (PrimeScript^TM^ RT Master Mix, RR036A, Applied, TaKaRa, Dalian, China). The quantitative real-time PCR (qRT-PCR) reaction was carried out with a quantitative real-time PCR master mix (RR390A, TaKaRa Bio Inc., Japan) in an ABI7500 Real-Time PCR system (Applied Biosystems, Foster City, CA, USA) in triplicate and repeated at least three times. β*-Actin* was used as an internal control. The relative expression of messenger RNA (mRNA) was calculated by the 2^−ΔΔCT^ method. The primer sequence and product size are shown in Table 3.

**Table 3:** Primer sequences used for quantitative real-time PCR (qRT-PCR).

**Target Genes**	**Primer sequence (5' to 3')**	**Product size**	**Accession no**.
***DMT1***	F:CTCAGCCATCGCCATCAACCT R:CTCCAGCTTCCGCAGACCATA	124	EF635922
***ZnT-1***	F:ATCTGCGAGTGCCTTCTTCCT R:ATGAACACTGATGGTAGCCTGGA	84	AJ619980
***FPN1***	F:GGAGACTGGGTGGACAAGAA R:GATGCATTCTGAACAACCAAGGA	67	NM_001012 913.1
***β-actin***	F:CGGTACCAATTACTGGTGTTAGATG R:GCCTTCATTCACATCTATCACTGG	134	NM_205518.1

### Western Blot Analysis

Duodenal protein was extracted according to the manufacturer's instructions for lysate (Beyotime, Shanghai, China). The Enhanced BCA Protein Assay Kit (Beyotime, Shanghai, China) was used to determine the concentration, and the protein content of each sample was adjusted to 50 μg. The proteins were separated by polyacrylamide gel electrophoresis and transferred to the immobilized protein transfer membrane (Solarbio, Beijing, China). After being washed three times with Western washing solution for 10 min each time and blocked using Protein Free Rapid Blocking Buffer (EpiZyme, Shanghai, China) for 20 min, the membranes were incubated with primary antibody: Anti-*DMT1* (1:500, bs-3577R, BIOSS, Beijing, China), Anti-*ZnT-1* (1:500, bs-6440R, BIOSS, Beijing, China), and Anti-SLC40A1/*FPN1* (1:500, 26601-1-AP, Proteintech, Wuhan, China) overnight. Membranes were incubated with Anti-Rabbit IgG (1:2500, Beyotime, Shanghai, China) diluted with secondary antibody diluent (Beyotime, Shanghai, China) at 37°C for 2 h. Then, they were soaked in high-sensitivity luminescent reagent (BeyoECL Plus, Beyotime, Shanghai, China) and quantified by image software (Image Pro Plus 6.0, Media Cybernetics, Silver Spring, MD, USA). All western blot experiments were conducted with three biological repeats and repeated three times. All results were normalized to β-actin and the data were expressed as the relative values to those of the control group.

### Statistical Analysis

All data were analyzed by using the general linear model (GLM) in SAS 9.4 (SAS Institute Inc., Cary, NC, USA), and differences among treatments were compared with Tukey's multiple range tests. Results were presented as the mean and standard error of the mean (SEM). All statements of significance are based on a probability of *p* < 0.05.

## Results

### Production Performance

The production performance of laying hens is shown in Table 4. There was no significant difference in average egg production rate, average egg weight, average daily feed intake, and feed/egg ratio between all groups.

**Table 4:** Effects of dietary organic trace elements supplementation on production performance of late-phase laying hens.

**Items**	**Control**	**OTE300**	**OTE450**	**OTE600**	**SEM**	***P*-value**
Average laying rate, %	83.63	83.60	84.27	82.72	0.229	0.118
Average egg weight, g	59.50	60.75	60.08	60.92	0.239	0.130
Average daily feed intake, g	138.64	138.66	139.46	138.78	0.143	0.136
Feed/egg ratio	2.34	2.28	2.28	2.28	0.010	0.163

### Egg Quality Analysis

The egg quality of laying hens is shown in Table 5. Compared with the control group, the eggshell strength in the OTE450 group increased dramatically (*p* < 0.05), while OTE300 and OTE600 did not affect eggshell strength. Nevertheless, dietary organic trace elements supplementation had no obvious effects on haugh unit, yolk weight, eggshell weight, and eggshell thickness compared with those in the control group.

**Table 5:** Effects of dietary organic trace elements supplementation on egg quality in late-phase laying hens.

**Items**	**Control**	**OTE300**	**OTE450**	**OTE600**	**SEM**	***P*-value**
Eggshell breaking strength, N	39.34^b^	38.32^b^	41.76^a^	37.75^b^	0.468	0.005
Haugh unit	65.55	73.38	68.15	62.33	2.042	0.281
Egg yolk weight, g	18.11	17.63	17.59	17.35	0.169	0.475
Eggshell weight, g	6.45	6.31	6.15	6.22	0.068	0.453
Eggshell thickness, mm	0.30	0.29	0.32	0.30	0.005	0.332

a, b *Means without a common letter differed, P < 0.05*.

### Serum and Feces Trace Element Analysis

As shown in Table 6, the serum copper concentration of the OTE450 group was the highest, while the content of the OTE300 group was the lowest (*p* < 0.05). There was no significant difference in serum copper concentration between the control and OTE600 groups. Moreover, iron and zinc concentrations have a similar trend with copper. However, the serum manganese concentration in the OTE450 group was significantly higher than that in the OTE300 group, but not significantly different from the OTE600 group and control group (*p* < 0.05). Trace elements deposition in the feces of laying hens is shown in Table 7. All of the trace elements (copper, iron, zinc, and manganese) had a lower deposition in the feces in organic trace element groups, compared with the control group (*p* < 0.05). With the increase in the amount of organic trace elements, the content of copper, iron, zinc, and manganese in feces increased significantly (*p* < 0.05).

**Table 6:** Effects of dietary organic trace elements supplementation on serum trace elements in late-phase laying hens (μg/ml).

**Items**	**Control**	**OTE300**	**OTE450**	**OTE600**	**SEM**	***P*-value**
Copper	1.53^b^	1.34^c^	1.64^a^	1.52^b^	0.026	<0.001
Iron	9.40^b^	8.45^c^	10.41^a^	9.65^b^	0.152	<0.001
Zinc	7.48^b^	6.52^c^	8.61^a^	7.05^b^	0.177	<0.001
Manganese	0.63^ab^	0.55^b^	0.69^a^	0.65^ab^	0.019	0.039

a−c *Means without a common letter differed, P < 0.05*.

**Table 7:** Effects of organic trace elements supplementation on feces trace elements in late-phase laying hens (mg/kg).

**Items**	**Control**	**OTE300**	**OTE450**	**OTE600**	**SEM**	***P*-value**
Copper	35.80^a^	24.48^d^	30.85^c^	33.83^b^	0.903	<0.001
Iron	1253.57^a^	967.29^d^	1003.91^c^	1063.98^b^	23.454	<0.001
Zinc	451.65^a^	266.52^d^	365.77^c^	388.55^b^	13.776	<0.001
Manganese	123.81^a^	89.17^d^	100.90^c^	115.90^b^	2.806	<0.001

a−d *Means without a common letter differed, P < 0.05*.

### Duodenal Morphology

**Figure 1:**
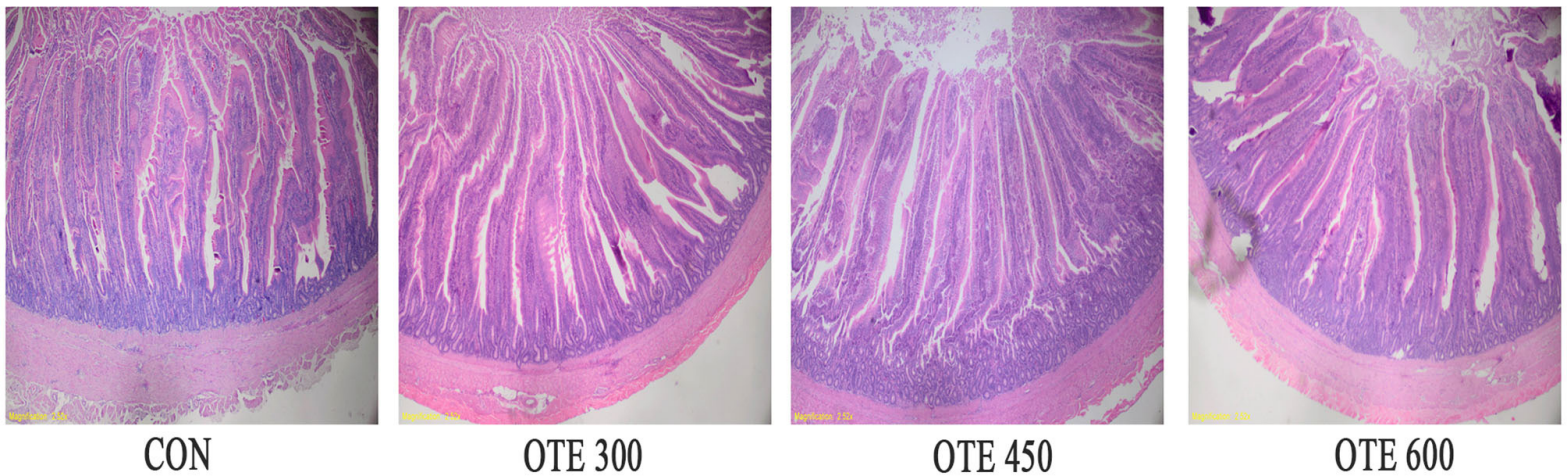
Effects of organic trace elements supplementation on duodenal morphology of late-phase laying hens. Representative images (×40) were stained with hematoxylin and eosin (H and E). Con represents control group, and OTE300, OTE450, and OTE600 represent the 300, and 600 mg/kg organic trace elements groups, respectively.

The effects of organic trace elements supplementation on the duodenal morphology of laying hens are shown in Table 8 and Figure 1. The supplementation of organic trace elements can significantly improve the villus height and villus concealment ratio (*p* < 0.05), among which the OTE450 group is the highest, the OTE600 group is the second, and there is no significant difference between the control group and OTE300 group.

**Table 8:** Effects of organic trace elements supplementation on duodenal morphology of late-phase laying hens.

**Items**	**Control**	**OTE300**	**OTE450**	**OTE600**	**SEM**	***P*-value**
Villus height, μm	1237.3^c^	1208.3^c^	1419.9^a^	1335.9^b^	26.785	<0.001
Crypt depth, μm	204.8	207.6	190.2	202.5	2.664	0.073
Velvet concealed ratio	6.0^c^	5.8^c^	7.5^a^	6.6^b^	0.203	<0.001

a−c *Means without a common letter differed, P < 0.05*.

### Expression of Trace Element Transporter

**Figure 2:**
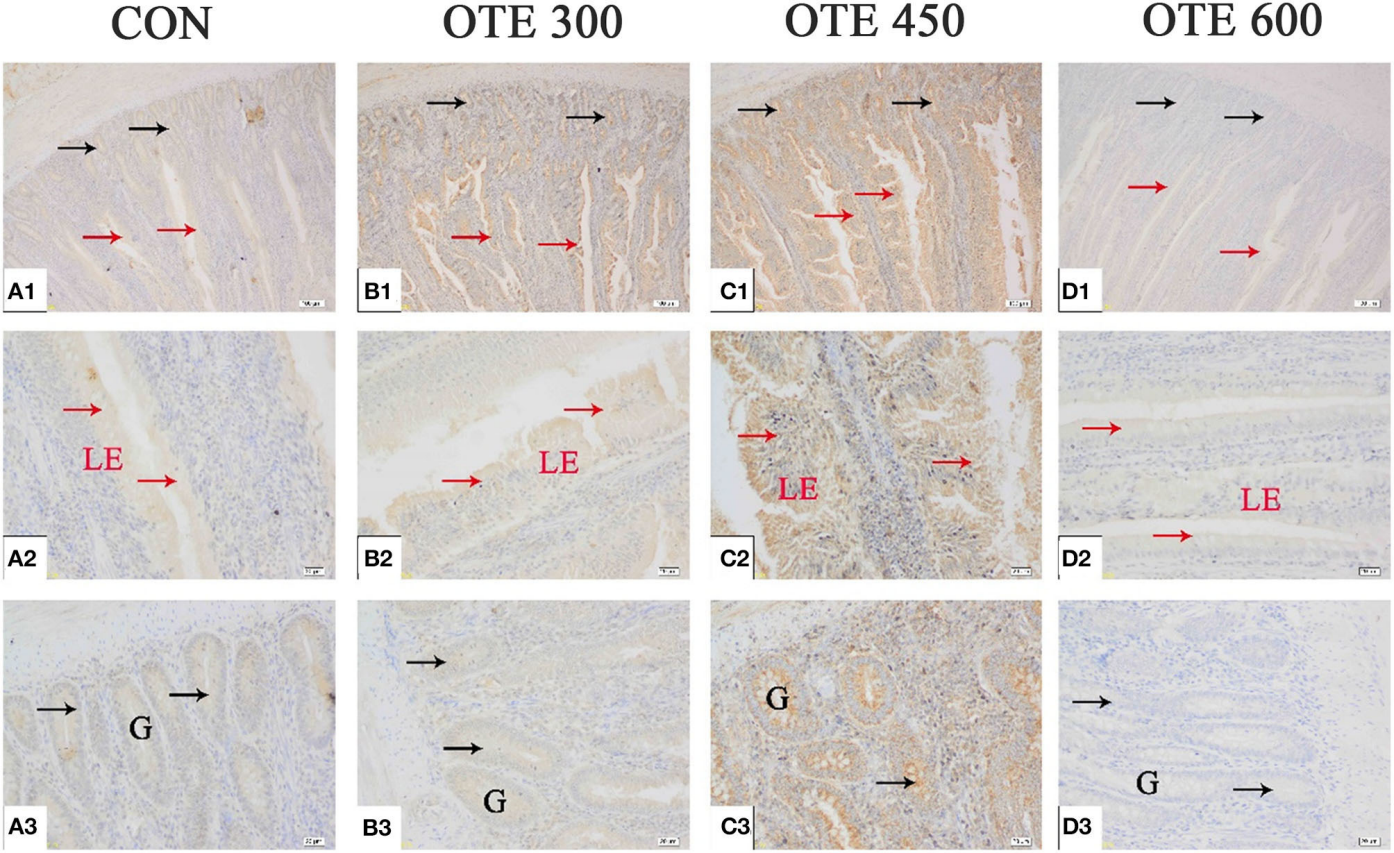
Effects of organic trace elements supplementation on divalent metal transporter (DMT1) localization in duodenum of late-phase laying hens. **(A–D)** Represent the control, 300, 450, and 600 mg/kg organic trace elements groups, respectively. Scale bars were 100 μm **(A1–D1)** and 20 μm **(A2–D2,A3–D3)**. The red arrow represents DMT1 immunopositive cells in the duodenal villi, and the black arrow represents DMT1 immunopositive cells in the duodenal gland (*n* = 6).

**Figure 3:**
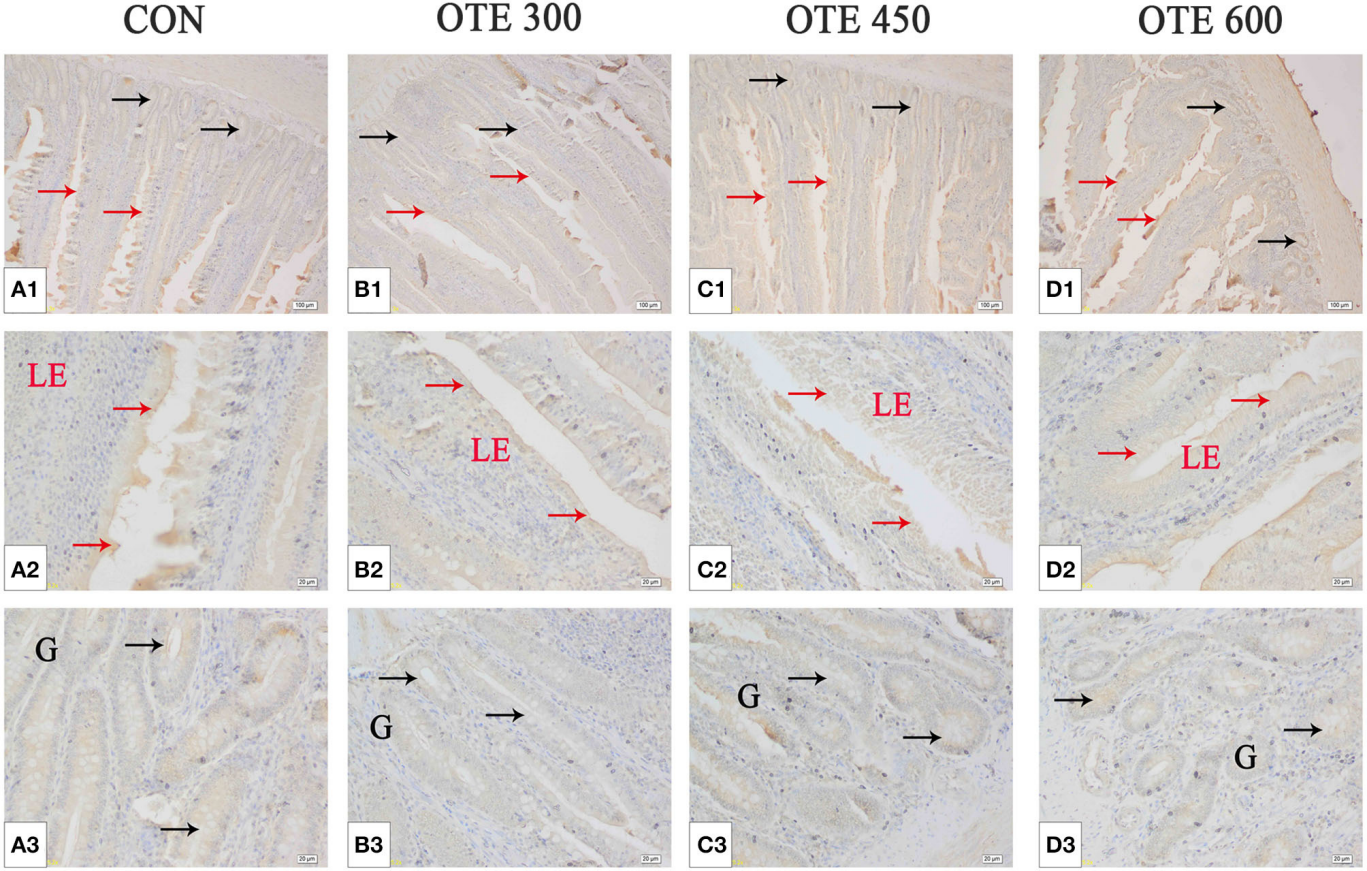
Effects of organic trace elements supplementation on zinc transporter 1 (ZnT-1) localization in duodenum of late-phase laying hens. **(A–D)** Represent the control, 300, 450, and 600 mg/kg organic trace elements, respectively. Scale bars were 100 μm **(A1–D1)** and 20 μm **(A2–D2,A3–D3)**. The red arrow represents ZnT-1 immunopositive cells in the duodenal villi, and the black arrow represents ZnT-1 immunopositive cells in the duodenal gland (*n* = 6).

**Figure 4:**
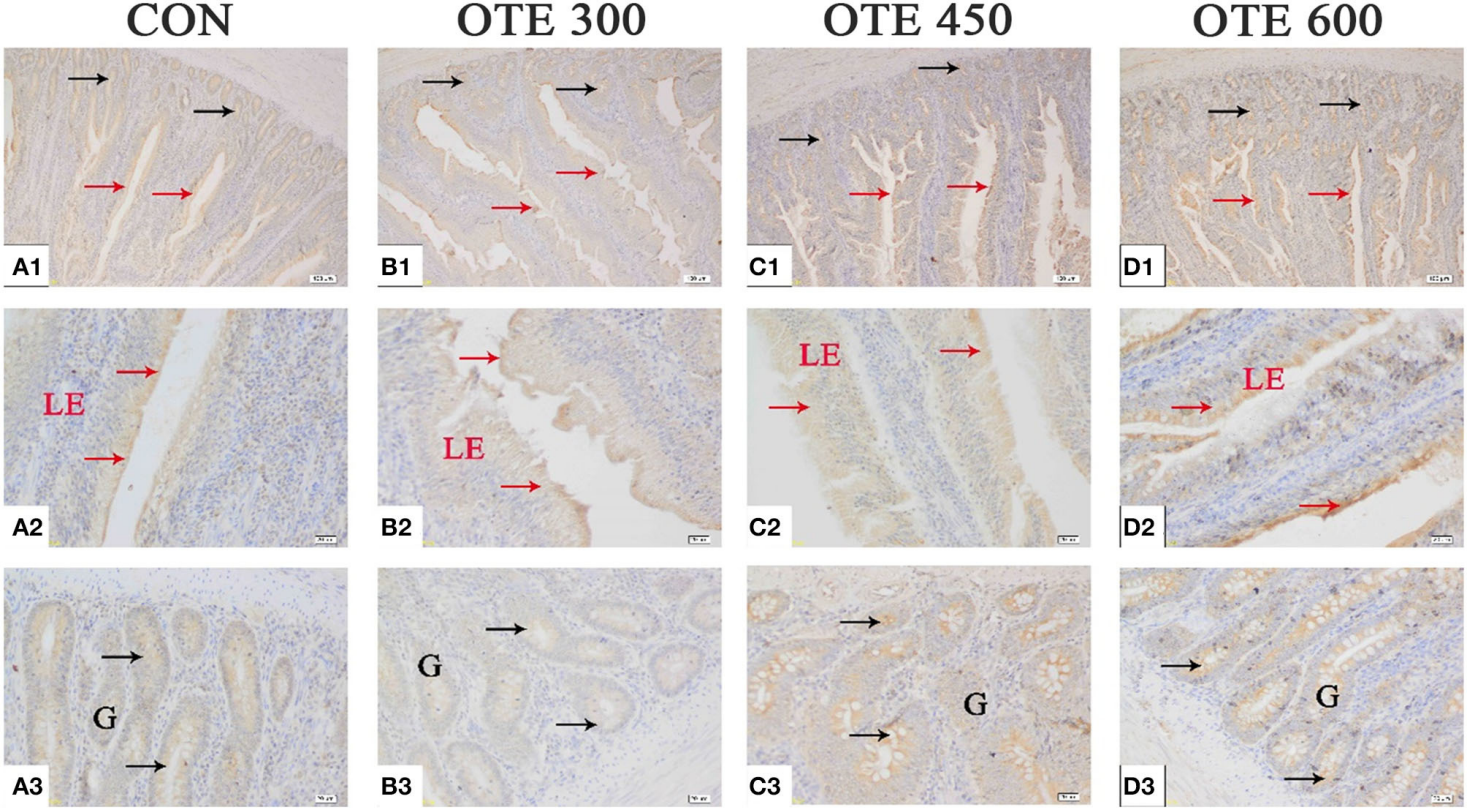
Effects of organic trace elements supplementation on ferroportin 1 (FPN1) localization in the duodenum of late-phase laying hens. **(A–D)** Represent the control, 300, 450, and 600 mg/kg organic trace elements, respectively. Scale bars were 100 μm **(A1–D1)** and 20 μm **(A2–D2,A3–D3)**. The red arrow represents FPN1 immunopositive cells in the duodenal villi, and the black arrow represents FPN1 immunopositive cells in the duodenal gland (*n* = 6).

**Figure 5:**
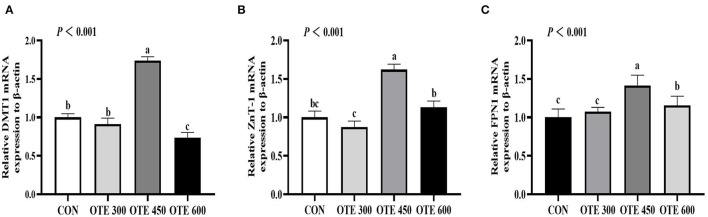
Effects of organic trace elements on messenger RNA (mRNA) expression of duodenal trace element transporter in late-phase laying hens. DMT1 **(A)**, divalent metal transporter 1; ZnT-1 **(B)**, zinc transporter 1; and FPN1 **(C)**, ferroportin 1. Different letters represent significant differences (*p* < 0.05).

**Figure 6:**
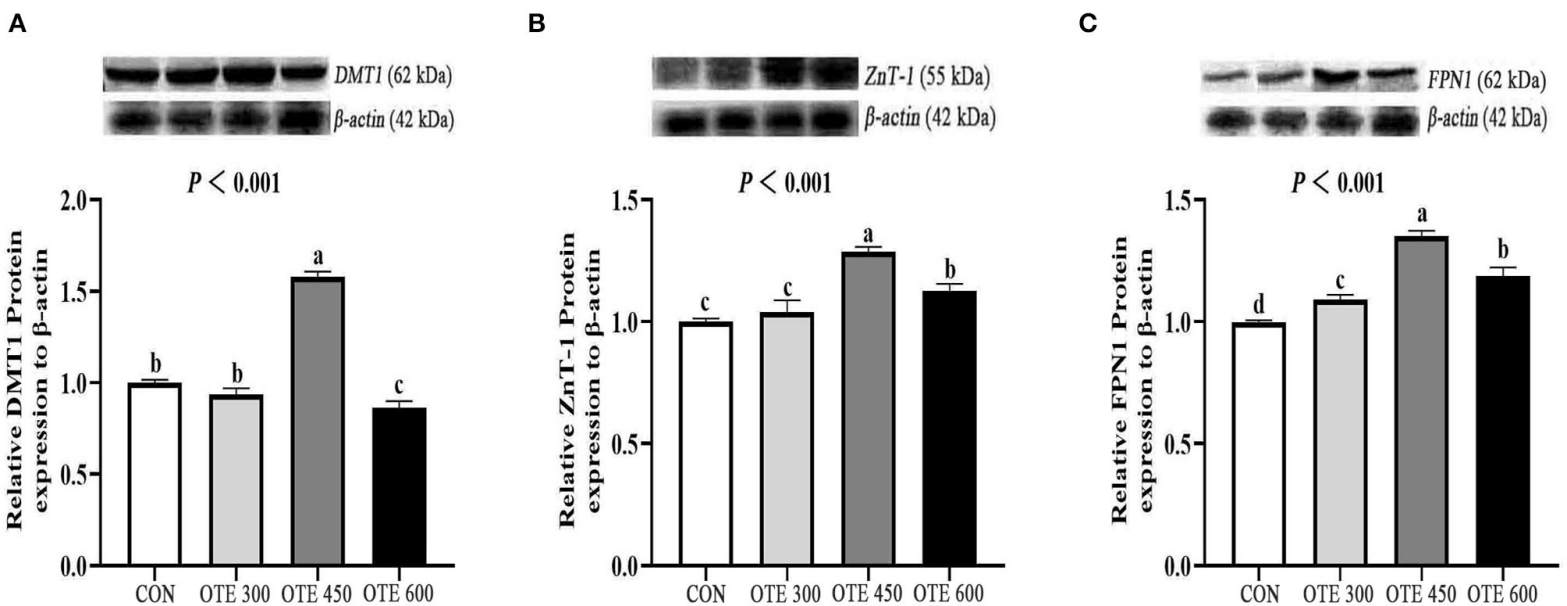
Efects of organic trace elements on protein expression of duodenal trace element transporter in late-phase laying hens. DMT1 **(A)**, divalent metal transporter 1; ZnT-1 **(B)**, zinc transporter; FPN1 **(C)**, ferroportin 1. Different letters represent significant differences (*p* < 0.05).

The immunohistochemical results of *DMT1, ZnT-1*, and *FPN1* in the duodenum are shown in Table 9 and Figures 2–4. The supplementation of organic trace elements increased the expression of *DMT1, ZnT-1*, and *FPN1* in villi and crypts (*p* < 0.05). The expression of *DMT1* in intestinal epithelial cells and crypts from high to low was OTE450 > OTE300 > control > OTE600 (*p* < 0.05). The expression of *ZnT-1* and *FPN1* in the control group was the lowest, followed by the OTE300 group, OTE450 group, and OTE600 group, but there was no significant difference between the three groups. The mRNA expressions of *DMT1, ZnT-1*, and *FPN1* in the layer duodenum are shown in Figure 5. The mRNA expression of *DMT1* in the OTE450 group was significantly higher than that in other groups (*p* < 0.05), and that in the OTE600 group was the lowest. The mRNA expression of *ZnT-1* in OTE450 was the highest, followed by that in OTE600 (*p* < 0.05), and then that in OTE300. The expression of *FPN1* was the highest in the OTE450 group (*p* < 0.05), followed by that in the OTE600 group, which was significantly higher than that in the control and OTE300 groups (*p* < 0.05). The protein expression of *DMT1, ZnT-1*, and *FPN1* in layer duodenum is shown in Figure 6. The expressions of *DMT1* and ZnT1 were higher in the OTE450 group than that in other groups (*p* < 0.05), but there was no significant difference between the OTE300 group and the control group, which were significantly higher than that in the OTE600 group. The expression relationship of *FPN1* protein was OTE450 > OTE600 > OTE300 > control.

**Table 9:** Effects of organic trace elements supplementation on cumulative optical density of divalent metal transporter 1 (DMT1), zinc transporter 1 (ZnT-1), and ferroportin 1 (FPN1) positive reactions in duodenum of late-phase laying hens (×10^3^).

**Items**	**Control**	**OTE300**	**OTE450**	**OTE600**	**SEM**	***P*-value**
**DMT1**
Duodenal villi	79.1^c^	94.3^b^	139.1^a^	39.5^d^	10.801	<0.001
Duodenal gland	72.2^c^	84.4^b^	138.5^a^	34.6^d^	11.239	<0.001
**ZnT-1**
Duodenal villi	88.7^c^	117.6^b^	145.6^a^	137.1^a^	6.181	<0.001
Duodenal gland	62.2^c^	75.4^b^	126.5^a^	127.6^a^	8.999	<0.001
**FPN1**
Duodenal villi	88.5^c^	116.9^b^	147.4^a^	134.1^a^	6.511	<0.001
Duodenal gland	67.9^c^	83.3^b^	115.8^a^	112.6^a^	6.146	<0.001

## Discussion

Copper, iron, zinc, and manganese are essential trace elements for the production performance, reproduction, and metabolism of laying hens. They play important roles in laying, tissue mineral element precipitation, and metabolism (19, 20). In a 57-week-old Beijing Red Layer hens experiment, replacing 0–50% inorganic trace elements with organic compound trace elements did not affect the growth performance of layers (9). In addition, the study showed that 50% NRC (18) level of organic mineral elements supplementation to 26 weeks Roman layer diets did not influence feed intake, egg-laying rate, and feed egg ratio (21). Similarly, some studies also found that organic trace elements supplementation had no significant effect on production performance compared with inorganic trace elements (22–24), which is consistent with the present study. However, there are some inconsistent reports with this study. The supplementation of 80 mg/kg zinc-methionine in 49-week-old layer diets significantly decreased the feed intake and increased egg production (25). In addition, the supplementation of organic trace elements could increase the average daily feed intake and egg production of 30-week-old layers under high temperatures (26). These inconsistencies may be caused by the breed and age of laying hens, and the form and level of trace elements in their diets.

Copper, iron, zinc, and manganese play important roles in the formation of eggs. Copper can catalyze the formation of eggshell membrane fiber structure through lysyl oxidase, and then increase the toughness of the eggshell membrane (27). Iron mainly affects eggshell color by regulating the formation and transport of protoporphyrin IX (28, 29). Zinc is an important part of bicarbonate, which can affect the formation of calcium carbonate in the shell gland by providing carbonate ions, and finally participate in the formation of eggshell (19). Manganese affects the activity of 1, 3-glucuronosyltransferase (GLcAT-I) by regulating galactose, thereby changing the quality of eggshells (30).

It has been reported that organic trace element supplementation can improve the eggshell strength of eggs (31, 32). In addition, organic trace elements were supplemented to a 47–62-week-old Hailan brown layer, which significantly improved the breaking strength and thickness of eggshells (33, 34). However, some studies reported that there was no significant difference in egg quality between organic trace elements and the inorganic trace elements group (8, 35). In this experiment, OTE450 supplementation significantly improved the strength of the eggshell but did not change the haugh unit, yolk weight, eggshell weight, and eggshell thickness. This is consistent with the study that the supplementation of organic trace elements promoted the fusion of the initial stage of the eggshell and improved the mechanical strength of the eggshell regardless of the thickness (32).

Organic trace elements have better bioavailability than inorganic trace elements. The supplementation of organic mineral elements has been proven to promote the absorption of trace elements and increase their levels in serum (36, 37). The supplementation of organic trace elements could significantly increase the serum contents of copper, iron, zinc, and manganese of 40-week-old broilers (38). Our results indicated that the content of serum trace elements was significantly increased by supplementing 450 mg/kg of organic trace elements. However, the contents of serum copper, iron, zinc, and manganese decreased in the OTE600 group. The reason for this phenomenon may be that the 450 mg/kg organic trace elements supplementation is the optimal level for maintaining the homeostasis of serum trace elements. More and less organic trace element supplementation could break this homeostasis, which results in the decrease of serum trace element content. Compared with inorganic trace elements, adding a lower level of organic trace elements can reduce the excretion of mineral elements in feces without having adverse effects on poultry health and production (39). Similar to our results, organic trace elements significantly reduced the contents of copper, iron, zinc, and manganese in feces, which increased linearly with the increase of organic trace elements level. Above all, organic trace elements can reduce the excretion of trace elements in feces by improving the absorption of mineral elements in serum, and the OTE450 group showed optimal effects.

Minerals were mainly absorbed by the duodenum and then transported to the portal vein circulation (40). Villus height and crypt depth can be used to evaluate intestinal development, digestion, and absorption capacity (41). The villus height and crypt depth represent the digestive and absorptive capacity of the small intestine and the maturity of epithelial cells, while the villi to crypt ratio represents the digestive and absorptive function of the small intestine (42). The supplementation of glycine chelated zinc can significantly improve the villus height and the ratio of the villus to the crypt in broilers (43). Our experiments showed that OTE450 and OTE600 significantly increased the villus height and the ratio of villus height to crypt depth compared with the control group. However, the villus height and ratio of villus height to crypt depth in the OTE600 group were lower than those in the OTE450 group. The phenomenon may be explained that compared with the OTE600 group, the OTE450 group has a better absorption capacity of trace elements. The study has shown that the increase in villus length is related to the digestion and absorption of nutrients (44, 45). Therefore, the villus height and ratio of villus height to crypt depth were higher in the OTE450 group.

Trace element transporters include *DMT1, ZnT-1*, and *FPN1*. *DMT1* can use a proton gradient to drive the absorption of divalent metal ions, such as copper, iron, and zinc (46). *DMT1* was found in the apical membrane of intestinal cells and slightly expressed in crypts (47). Trace elements could be transported by *DMT1* to small intestinal epithelial cells through microvilli (48, 49). *FPN1* is the only iron transporter in the intestinal tract in animals, which is located in the basal layer of duodenal intestinal cells to transport iron and manganese to blood (50–53). Similarly, as a zinc transporter, *ZnT-1* is widely expressed in the duodenal basement membrane and functions to transport zinc from intestinal epithelial cells to the blood. It was found that the mRNA expression of *DMT1* in broilers fed with organic manganese was significantly increased (54). Additionally, the supplementation of organic manganese significantly increased the mRNA and protein expression of *DMT1* and *FPN1* in 15-day-old broilers (55). Moreover, a report found that the mRNA expression of *FPN1* was significantly increased in 10-day-old layers fed with manganese (56). In addition, studies showed that zinc supplementation could increase the mRNA expression of *ZnT-1* in chicken, Caco-2 cells, and rat small intestine (57–59). In this study, the mRNA and protein expression of duodenal *DMT1, ZnT-1*, and *FPN1* increased in the organic trace element group, indicating that a low level of organic trace elements could promote the absorption and excretion of divalent metal ions in intestinal epithelial cells. In addition, the IOD value of IHC showed that the supplementation of organic trace elements significantly increased the expression of *DMT1, ZnT-1*, and *FPN1* in the duodenum. This is consistent with the results of western blot analysis.

## Conclusions

In conclusion, the addition of 450 mg/kg of organic trace elements to the diet significantly improved the eggshell strength and serum trace element deposition of laying hens without affecting the production performance, increased the height of duodenal villi and the ratio of villi height to crypt depth, as well as the mRNA and protein expression of *DMT1, ZnT-1*, and *FPN1*. This indicates that low levels of organic trace elements can improve eggshell strength, trace element utilization, and duodenal function.

## Data Availability Statement

The original contributions presented in the study are included in the article/supplementary material, further inquiries can be directed to the corresponding author/s.

## Ethics Statement

The animal study was reviewed and approved by the Committee on the Ethics of Shandong Agricultural University. Written informed consent was obtained from the owners for the participation of their animals in this study.

## Author Contributions

W-rY and S-zJ received funding. NJ and YL conceptualized and designed the study. XC and FZ conducted animal experiments, chemical analyses, and analyzed the data. XC wrote the original. NJ, S-zJ, and L-bH reviewed and revised the draft. All authors agreed to publish the manuscript.

## Funding

This research was supported by the Major Innovative Projects of Shandong Province (grant number 2019JZZY020609).

## Conflict of Interest

The authors declare that the research was conducted in the absence of any commercial or financial relationships that could be construed as a potential conflict of interest.

## Publisher's Note

All claims expressed in this article are solely those of the authors and do not necessarily represent those of their affiliated organizations, or those of the publisher, the editors and the reviewers. Any product that may be evaluated in this article, or claim that may be made by its manufacturer, is not guaranteed or endorsed by the publisher.

## References

[1] Ramos-Vidales D Gómez-Verduzco G Cortes-Cuevas A Del Río-García JC Fernández-Tinoco S Chárraga-Aguilar S. Organic trace minerals on productive performance, egg quality and immune response in Bovans White laying hens. J Anim Physiol Anim Nutr. (2019) 103:1484–91. 10.1111/jpn.13156 31350792

[2] Echeverry H Yitbarek A Munyaka P Alizadeh M Cleaver A Camelo-Jaimes G. Organic trace mineral supplementation enhances local and systemic innate immune responses and modulates oxidative stress in broiler chickens. Poult Sci. (2016) 95:518–27. 10.3382/ps/pev374 26740133

[3] Brugger D Windisch WM. Environmental responsibilities of livestock feeding using trace mineral supplements. Anim Nutr. (2015) 1:113–8. 10.1016/j.aninu.2015.08.005 29767146 PMC5945946

[4] Jarosz L Marek A Gradzki Z Kwiecien M Zylinska B Kaczmarek B. Effect of feed supplementation with zinc glycine chelate and zinc sulfate on cytokine and immunoglobulin gene expression profiles in chicken intestinal tissue. Poult Sci. (2017) 96:4224–35. 10.3382/ps/pex253 29053834

[5] Bai SP Lu L Luo XG Liu B. Kinetics of manganese absorption in ligated small intestinal segments of broilers. Poult Sci. (2008) 87:2596–604. 10.3382/ps.2008-00117 19038816

[6] Yenice E Mizrak C Gültekin M Atik Z Tunca M. Effects of organic and inorganic forms of manganese, zinc, copper, and chromium on bioavailability of these minerals and calcium in late-phase laying hens. Biol Trace Elem Res. (2015) 167:300–7. 10.1007/s12011-015-0313-8 25800653

[7] Muszyński S Tomaszewska E Kwiecień M Dobrowolski P Tomczyk A. Effect of dietary phytase supplementation on bone and hyaline cartilage development of broilers fed with organically complexed copper in a cu-deficient diet. Biol Trace Elem Res. (2018) 182:339–53. 10.1007/s12011-017-1092-1 28710591 PMC5838127

[8] Gheisari AA Sanei A Samie A Gheisari MM Toghyani M. Effect of diets supplemented with different levels of manganese, zinc, and copper from their organic or inorganic sources on egg production and quality characteristics in laying hens. Biol Trace Elem Res. (2011) 142:557–71. 10.1007/s12011-010-8779-x 20711683

[9] Zhang KK Han MM Dong YY Miao ZQ Zhang JZ Song XY. Low levels of organic compound trace elements improve the eggshell quality, antioxidant capacity, immune function, and mineral deposition of aged laying hens. Animal. (2021) 15:100401. 10.1016/j.animal.2021.100401 34794097

[10] Jarosz ŁS Marek A Gradzki Z Kwiecień M Kaczmarek B. The effect of feed supplementation with a copper-glycine chelate and copper sulphate on selected humoral and cell-mediated immune parameters, plasma superoxide dismutase activity, ceruloplasmin and cytokine concentration in broiler chickens. J Anim Physiol Anim Nutr. (2018) 102:e326–36. 10.1111/jpn.12750 28603872

[11] Zhang YN Wang J Zhang HJ Wu SG Qi GH. Effect of dietary supplementation of organic or inorganic manganese on eggshell quality, ultrastructure, and components in laying hens. Poult Sci. (2017) 96:2184–93. 10.3382/ps/pew495 28204746

[12] Xiao JF Wu SG Zhang HJ Yue HY Wang J Ji F. Bioefficacy comparison of organic manganese with inorganic manganese for eggshell quality in Hy-Line Brown laying hens. Poult Sci. (2015) 94:1871–8. 10.3382/ps/pev138 26047673

[13] Qiu JL Zhou Q Zhu JM Lu XT Liu B Yu DY. Organic trace minerals improve eggshell quality by improving the eggshell ultrastructure of laying hens during the late laying period. Poult Sci. (2020) 99:1483–90. 10.1016/j.psj.2019.11.006 32115033 PMC7587740

[14] Umar Yaqoob M Wang G Sun W Pei X Liu L Tao W. Effects of inorganic trace minerals replaced by complexed glycinates on reproductive performance, blood profiles, and antioxidant status in broiler breeders. Poult Sci. (2020) 99:2718–26. 10.1016/j.psj.2019.11.058 32359609 PMC7597384

[15] Sirri F Zampiga M Berardinelli A Meluzzi A. Variability and interaction of some egg physical and eggshell quality attributes during the entire laying hen cycle. Poult Sci. (2018) 97:1818–23. 10.3382/ps/pex456 29506193

[16] Bai S Huang L Luo Y Wang L Ding X Wang J. Dietary manganese supplementation influences the expression of transporters involved in iron metabolism in chickens. Biol Trace Elem Res. (2014) 160:352–60. 10.1007/s12011-014-0073-x 25037067

[17] Zhang LY Li XF Liao XD Zhang LY Lu L Luo XG. Effect of iron source on iron absorption and gene expression of iron transporters in the ligated duodenal loops of broilers. J Anim Sci. (2017) 95:1587–97. 10.2527/jas.2016.1147 28464091

[18] NRC. National Research Council: Nutrient Requirements of Poultry. Washington, DC: NRC (1994).

[19] Nys Y Schlegel P Durosoy S. Adapting trace mineral nutrition of birds for optimising the environment and poultry product quality. Worlds Poult Sci J. (2018) 74:225–38. 10.1017/S0043933918000016

[20] Dobrzañski Z Korczyñski M Chojnacka K Górecki H Opaliñski S. Influence of organic forms of copper, manganese and iron on bioaccumulation of these metals and zinc in laying hens. J Elementol. (2008) 13:309–19. 10.2112/08A-0006.1

[21] Yang K Hu S Mu R Qing Y Xie L Zhou L. Effects of different patterns and sources of trace elements on laying performance, tissue mineral deposition, and fecal excretion in laying hens. Animals. (2021) 11:1164. 10.3390/ani11041164 33921551 PMC8072985

[22] Tabatabaie MM Aliarabi H Saki AA Ahmadi A Siyar SA. Effect of different sources and levels of zinc on egg quality and laying hen performance. Pak J Biol Sci. (2007) 10:3476–8. 10.3923/pjbs.2007.3476.3478 19090175

[23] Behjatian E sfahani M Moravej H Ghaffarzadeh M Nehzati-Paghaleh GA. Comparison the Zn-threonine, Zn-methionine, and Zn oxide on performance, egg quality, Zn bioavailability, and Zn content in egg and excreta of laying hens. Biol Trace Elem Res. (2021) 199:292–304. 10.1007/s12011-020-02141-8 32367378

[24] Cufadar Y Göçmen R Kanbur G Yildirim B. Effects of dietary different levels of nano, organic and inorganic zinc sources on performance, eggshell quality, bone mechanical parameters and mineral contents of the tibia, liver, serum and excreta in laying hens. Biol Trace Elem Res. (2020) 193:241–51. 10.1007/s12011-019-01698-3 30941677

[25] Li LL Gong YJ Zhan HQ Zheng YX Zou XT. Effects of dietary Zn-methionine supplementation on the laying performance, egg quality, antioxidant capacity, and serum parameters of laying hens. Poult Sci. (2019) 98:923–31. 10.3382/ps/pey440 30299460

[26] Saleh AA Eltantawy MS Gawish EM Younis HH Amber KA Ebeid TA. Impact of dietary organic mineral supplementation on reproductive performance, egg quality characteristics, lipid oxidation, ovarian follicular development, and immune response in laying hens under high ambient temperature. Biol Trace Elem Res. (2020) 195:506–14. 10.1007/s12011-019-01861-w 31418151

[27] Baumgartner S Brown DJ Salevsky E Jr Leach RM Jr. Copper deficiency in the laying hen. J Nutr. (1978) 108:804–11. 10.1093/jn/108.5.804 641596

[28] Bi H Liu Z Sun C Li G Wu G Shi F. Brown eggshell fading with layer ageing: dynamic change in the content of protoporphyrin IX. Poult Sci. (2018) 97:1948–53. 10.3382/ps/pey044 29509933

[29] Gao G Li J Zhang Y. Cellular iron metabolism and regulation. Adv Exp Med Biol. (2019) 21–32. 10.1007/978-981-13-9589-5_2 31456203

[30] Xiao JF Zhang YN Wu SG Zhang HJ Yue HY Qi GH. Manganese supplementation enhances the synthesis of glycosaminoglycan in eggshell membrane: a strategy to improve eggshell quality in laying hens. Poult Sci. (2014) 93:380–8. 10.3382/ps.2013-03354 24570460

[31] Manangi MK Vazques-Anon M Richards JD. The impact of feeding supplemental chelated trace minerals on shell quality, tibia breaking strength, and immune response in laying hens. J Appl Poult Res. (2015) 24:316–26. 10.3382/japr/pfv029

[32] Mabe I Rapp C Bain MM Nys Y. Supplementation of a corn-soybean meal diet with manganese, copper, and zinc from organic or inorganic sources improves eggshell quality in aged laying hens. Poult Sci. (2003) 82:1903–13. 10.1093/ps/82.12.1903 14717548

[33] Stefanello C Santos TC Murakami AE Martins EN Carneiro TC. Productive performance, eggshell quality, and eggshell ultrastructure of laying hens fed diets supplemented with organic trace minerals. Poult Sci. (2014) 93:104–13. 10.3382/ps.2013-03190 24570429

[34] Zhang YN Zhang HJ Wang J Yue HY Qi XL Wu SG. Effect of dietary supplementation of organic or inorganic zinc on carbonic anhydrase activity in eggshell formation and quality of aged laying hens. Poult Sci. (2017) 96:2176–83. 10.3382/ps/pew490 28204703

[35] Molnár A Maertens L Ampe B Buyse J Kempen I Zoons J. Changes in egg quality traits during the last phase of production: is there potential for an extended laying cycle *Br*. Poult Sci. (2016) 57:842–7. 10.1080/00071668.2016.1209738 27385085

[36] Abdallah AG El-Husseiny OM Abdel-Latif KO. Influence of some dietary organic mineral supplementations. Int J Poult Sci. (2009) 8:291–8. 10.3923/ijps.2009.291.29821912962

[37] Yu Q Liu H Yang K Tang X Chen S Ajuwon KM. Effect of the level and source of supplementary dietary zinc on egg production, quality, and zinc content and on serum antioxidant parameters and zinc concentration in laying hens. Poult Sci. (2020) 99:6233–8. 10.1016/j.psj.2020.06.029 33142541 PMC7647701

[38] Wang G Liu L Wang Z Pei X Tao W Xiao Z. Comparison of inorganic and organically bound trace minerals on tissue mineral deposition and fecal excretion in broiler breeders. Biol Trace Elem Res. (2019) 189:224–32. 10.1007/s12011-018-1460-5 30062463

[39] Vieira R Ferket P Malheiros R Hannas M Crivellari R Moraes V. Feeding low dietary levels of organic trace minerals improves broiler performance and reduces excretion of minerals in litter. Br Poult Sci. (2020) 61:574–82. 10.1080/00071668.2020.1764908 32362137

[40] Fish EM Burns B. Physiolog Small Bowel. Treasure Island, FL: StatPearls Publishing (2022). 30335296

[41] Missotten JA Michiels J Dierick N Ovyn A Akbarian A De Smet S. Effect of fermented moist feed on performance, gut bacteria and gut histo-morphology in broilers. Br Poult Sci. (2013) 54:627–34. 10.1080/00071668.2013.811718 23927009

[42] Xu FZ Zeng XG Ding XL. Effects of replacing soybean meal with fermented rapeseed meal on performance, serum biochemical variables and intestinal morphology of broilers. Asian Australas J Anim Sci. (2012) 25:1734–41. 10.5713/ajas.2012.12249 25049539 PMC4094158

[43] De Grande A Leleu S Delezie E Rapp C De Smet S Goossens E. Dietary zinc source impacts intestinal morphology and oxidative stress in young broilers. Poult Sci. (2020) 99:441–53. 10.3382/ps/pez525 32416829 PMC7587869

[44] Awad WA Hess C Hess M. Enteric pathogens and their toxin-induced disruption of the intestinal barrier through alteration of tight junctions in chickens. Toxins. (2017) 9:60. 10.3390/toxins9020060 28208612 PMC5331439

[45] Fan W Shi J Wang B Zhang M Kong M Li W. Effects of zinc and *Bacillus subtilis* on the reproductive performance, egg quality, nutrient digestion, intestinal morphology, and serum antioxidant capacity of geese breeders. Poult Sci. (2022) 101:101677. 10.1016/j.psj.2021.101677 35051674 PMC8883061

[46] Bai S Cao S Ma X Li X Liao X Zhang L. Organic iron absorption and expression of related transporters in the small intestine of broilers. Poult Sci. (2021) 100:101182. 10.1016/j.psj.2021.101182 34198093 PMC8253913

[47] Morgan EH Oates PS. Mechanisms and regulation of intestinal iron absorption. Blood Cells Mol Dis. (2002) 29:384–99. 10.1006/bcmd.2002.0578 12547229

[48] Hundal HS Taylor PM. Amino acid transceptors: gate keepers of nutrient exchange and regulators of nutrient signaling. Am J Physiol Endocrinol Metab. (2009) 296:E603–13. 10.1152/ajpendo.91002.2008 19158318 PMC2670634

[49] Espinoza A Le Blanc S Olivares M Pizarro F Ruz M Arredondo M. Iron, copper, and zinc transport: inhibition of divalent metal transporter 1 (DMT1) and human copper transporter 1 (hCTR1) by shRNA. Biol Trace Elem Res. (2012) 146:281–6. 10.1007/s12011-011-9243-2 22068728

[50] Canonne-Hergaux F Donovan A Delaby C Wang HJ Gros P. Comparative studies of duodenal and macrophage ferroportin proteins. Am J Physiol Gastrointest Liver Physiol. (2006) 290:G156–63. 10.1152/ajpgi.00227.2005 16081760

[51] Laftah AH Sharma N Brookes MJ McKie AT Simpson RJ Iqbal TH. Tumour necrosis factor alpha causes hypoferraemia and reduced intestinal iron absorption in mice. Biochem J. (2006) 397:61–7. 10.1042/BJ20060215 16566752 PMC1479761

[52] Abboud S Haile DJ. A novel mammalian iron-regulated protein involved in intracellular iron metabolism. J Biol Chem. (2000) 275:19906–12. 10.1074/jbc.M000713200 10747949

[53] Garcia SJ Gellein K Syversen T Aschner M. A manganese-enhanced diet alters brain metals and transporters in the developing rat. Toxicol Sci. (2006) 92:516–25. 10.1093/toxsci/kfl017 16705042

[54] Bai SP Lu L Wang RL Xi L Zhang LY Luo XG. Manganese source affects manganese transport and gene expression of divalent metal transporter 1 in the small intestine of broilers. Br J Nutr. (2012) 108:267–76. 10.1017/S0007114511005629 22172096

[55] Liao XD Wang G Lu L Zhang LY Lan YX Li SF. Effect of manganese source on manganese absorption and expression of related transporters in the small intestine of broilers. Poult Sci. (2019) 98:4994–5004. 10.3382/ps/pez293 31135902

[56] Meng T Gao L Xie C Xiang Y Huang Y Zhang Y. Manganese methionine hydroxy analog chelated affects growth performance, trace element deposition and expression of related transporters of broilers. Anim Nutr. (2021) 7:481–7. 10.1016/j.aninu.2020.09.005 34258436 PMC8245798

[57] McMahon RJ Cousins RJ. Regulation of the zinc transporter ZnT-1 by dietary zinc. Proc Natl Acad Sci. (1998) 95:4841–6. 10.1073/pnas.95.9.4841 9560190 PMC20175

[58] Liuzzi JP Blanchard RK Cousins RJ. Differential regulation of zinc transporter 1, 2, and 4 mRNA expression by dietary zinc in rats. J Nutr. (2001) 131:46–52. 10.1093/jn/131.1.46 11208937

[59] Iyengar V Pullakhandam R Nair KM. Coordinate expression and localization of iron and zinc transporters explain iron-zinc interactions during uptake in Caco-2 cells: implications for iron uptake at the enterocyte. J Nutr Biochem. (2012) 23:1146–54. 10.1016/j.jnutbio.2011.06.008 22137264

